# Case report: COPA syndrome with interstitial lung disease, skin involvement, and neuromyelitis spectrum disorder

**DOI:** 10.3389/fped.2023.1118097

**Published:** 2023-03-09

**Authors:** Xiao Li, Yu Tang, Lei Zhang, Yuan Wang, Weihua Zhang, Ying Wang, Yuelin Shen, Xiaolei Tang

**Affiliations:** ^1^ Department of Respiratory Medicine, Children’s Hospital Affiliated to Zhengzhou University/Henan Children’s Hospital/Zhengzhou Children’s Hospital, Zhengzhou, China; ^2^ Department of Neurology, Children’s Hospital Affiliated to Zhengzhou University/Henan Children’s Hospital/Zhengzhou Children’s Hospital, Zhengzhou, China; ^3^ Department of Neurology, Beijing Children’s Hospital, Capital Medical University, National Center for Children’s Health, Beijing, China; ^4^ Department of Respiratory Medicine, Beijing Children’s Hospital, Capital Medical University, National Center for Children’s Health, Beijing, China

**Keywords:** children, copa syndrome, neuromyelitis optica spectrum disorder, rashes, sirolimus

## Abstract

This report describes a case of a 22 months Chinese boy with COPA syndrome bearing the c.715G > C (p.A239P) genotype. In addition to interstitial lung diseae, he also suffered from recurrent chilblain-like rashes, which has not been previously reported, and neuromyelitis optica spectrum disorder (NMOSD), which is a very rare phenotype. Clinical manifestations expanded the phenotype of COPA syndrome. Notably, there is no definitive treatment for COPA syndrome. In this report, the patient has achieved short-term clinical improvement with sirolimus.

## Introduction

In 2019, the International Union of Immunological Societies Expert Committee (IUIS) classified COPA syndrome as a non-inflammasome-associated disease (1). COPA syndrome shares very similar symptoms and pathogenesis with another Type I interferonopathy, namely, STING-associated vasculopathy with onset in infancy (SAVI). STING and subsequent IFN pathway are activated by the mutation of *STING1* gene. Up till now, a total of 78 individuals carrying 16 variants of *COPA* gene have been reported in the literature. COPA syndrome, also known as an immune deficiency disease with strong clinical heterogeneity and certain commonality (2), usually affects the lungs, kidneys, and joints. Skin vasculopathy is considered as a core feature of SAVI, which was observed in 77% of the reported patients, but has yet to be described in any patient with COPA syndrome. In this report, we present a pediatric case with recurrent chilblain-like rashes, which is a kind of skin vasculopathy. In addition, the patient also suffered from NMOSD, which has been reported only once before.

## Case presentation

**Figure 1:**
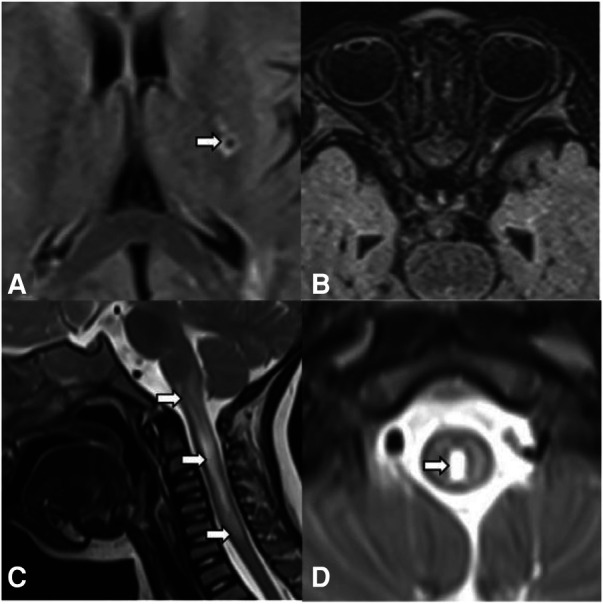
Brain MRI, spinal cord MRI, and optic MRI of the patient. Axial T2-weighted fluid-attenuated inversion recovery (FLAIR) MRI shows small encephalomalacia foci with gliosis in the left basal ganglia (**A**, arrows) and normal optic nerves (**B**). Sagittal (**C**) and axial (**D**) T2-weighted MRI of the spinal cord demonstrates longitudinally extensive transverse myelitis (LETM) lesion involving medulla to C6 leve (arrows).

A 22-month-old boy presented with irritability for 8 days and limb weakness for 6 days, accompanied by lethargy and vomiting. The boy had a history of transient paralysis of the right upper limb after fever at the age of 19-month-old, and his symptoms alleviated three days later. Additionally, he also manifested repeated facial and ear rashes, which were diagnosed as chilblain. Physical examination showed decreased muscle strength (3+/5 and 3/5 for upper and lower limbs, respectively), mild hypertonia in the left lower limb, nuchal rigidity, normal deep reflex, negative Babinski's sign, Kernig's sign and Brudzinski's sign. Laboratory tests (Table 1) revealed positive perinuclear antineutrophil cytoplasmic antibody (pANCA) and antinuclear antibody (ANA) (1:320), negative anti-double-stranded DNA (ds-DNA) antibody and anti-Sm antibody, positive serum anti-aquaporin 4 antibody (AQP4) (1:1,000, cell based assay), negative serum anti-myelin oligodendrocyte glycoprotein antibody (MOG), anti-glial fibrillary acidic protein (GFAP) antibody and serum antibodies against autoimmune peripheral neuropathy (KingMed Diagnostics). No abnormalities was found in ophthalmic examination revealed. Routine and biochemical test of cerebrospinal fluid (CSF) were normal. CSF culture and metagenomic next-generation sequencing (mNGS) showed no etiology. Brain MRI showed lesions involving medulla and white matter around the ventricle and small encephalomalacia foci with gliosis in the left basal ganglia (Figure 1A). MRI of the optic nerve was normal (Figure 1B). MRI of the Spinal cord showed longitudinally extensive transverse myelitis lesions from the medulla to C6 level (Figures 1C,D). According to the NMOSD diagnostic criteria proposed by the NMO diagnostic Group in 2015 (2), the child was diagnosed as NMOSD (based on the existence of two core clinical symptoms, namely, acute myelitis and area postrema syndrome, and positive AQP4 antibody). The patient was reated with high dose of intravenous methylprednisolone (20 mg per kilogram for 4 day) followed by oral prednisone with gradual diminution of dose, along with intravenous immunogloblin (IVIG) (2 g/kg). Then his muscle strength gradually improved without recurrence. Disease-Modifying Treatment (DMT) is recommended as the standard treatment in remission of NMODS, which can reduce the clinical onset of patients with recurrent remission. Regrettably, his parents refused to use DMT in consideration of its adverse effects.

**Table 1:** Clinical data of the patient with COPA syndrome.

Clinical data	
Sex	male
Age (m)	22
History	transient paralysis of the right upper limb
Symptom/sign	cough; tachypnea; clubbing; Chilblain - like rash; NMOSD
Lung	
CT chest	ground glass opacities (GGO); consolidations
TBFV	Mixed
Arthritis	–
Kidney	–
Allergic disease	Urticaria
Others	–
ANA	+ (1:320)
dsDNA	–
RF	+
HLA-B27	–
Anti-CCP	–
MPO	–
PR3	–
CRP (mg/L)	0.49 (0–10)
ESR (mm/h)	20 (1–15)
IL-6 (pg/ml)	3.4 (<7)
Total T lymphocytes	994.01 (770–2,860)
AQP4	+(1:1,000)

TBFV, tidal breathing flow volume curve; ANA, antinuclear antibody; CCP, cyclic citrullinated peptide; CRP, C-reactive protein; ESR, erythrocyte sedimentation rate; PFT, pulmonary function test; AQP4, anti-aquaporin 4 antibody.

**Figure 2:**
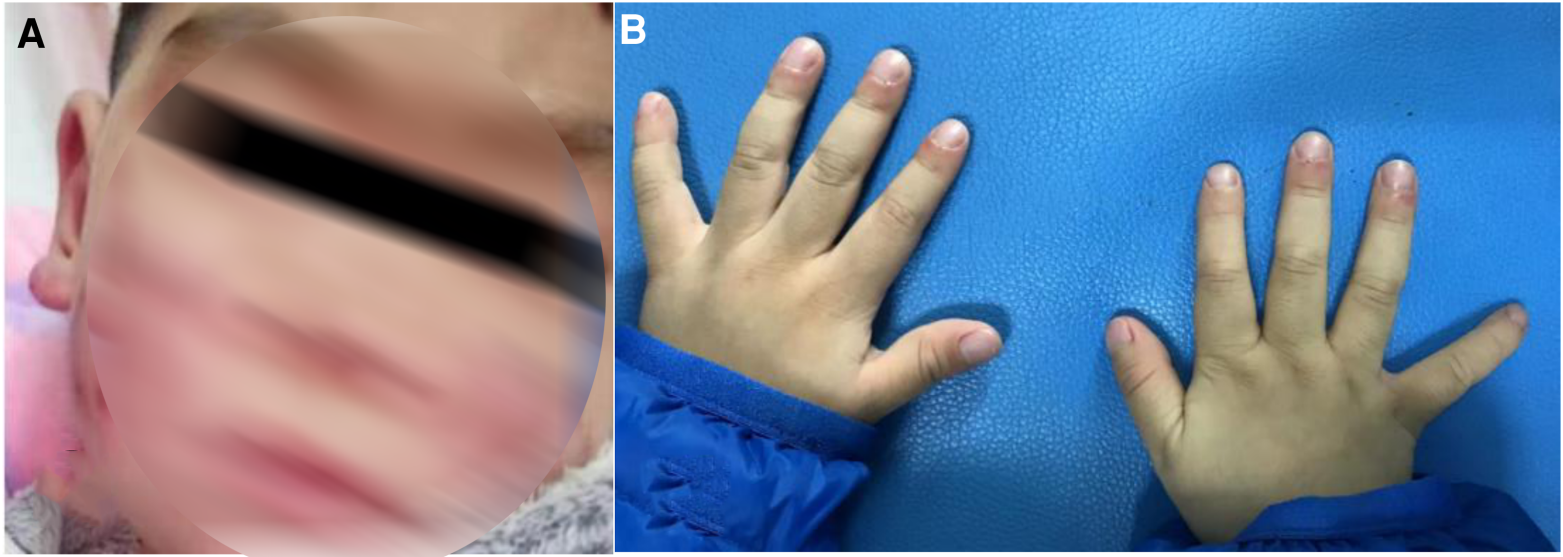
(**A**) Chilblain-like rash was observed on the face and ears of the child. (**B**) Clubbing fingers.

**Figure 3:**
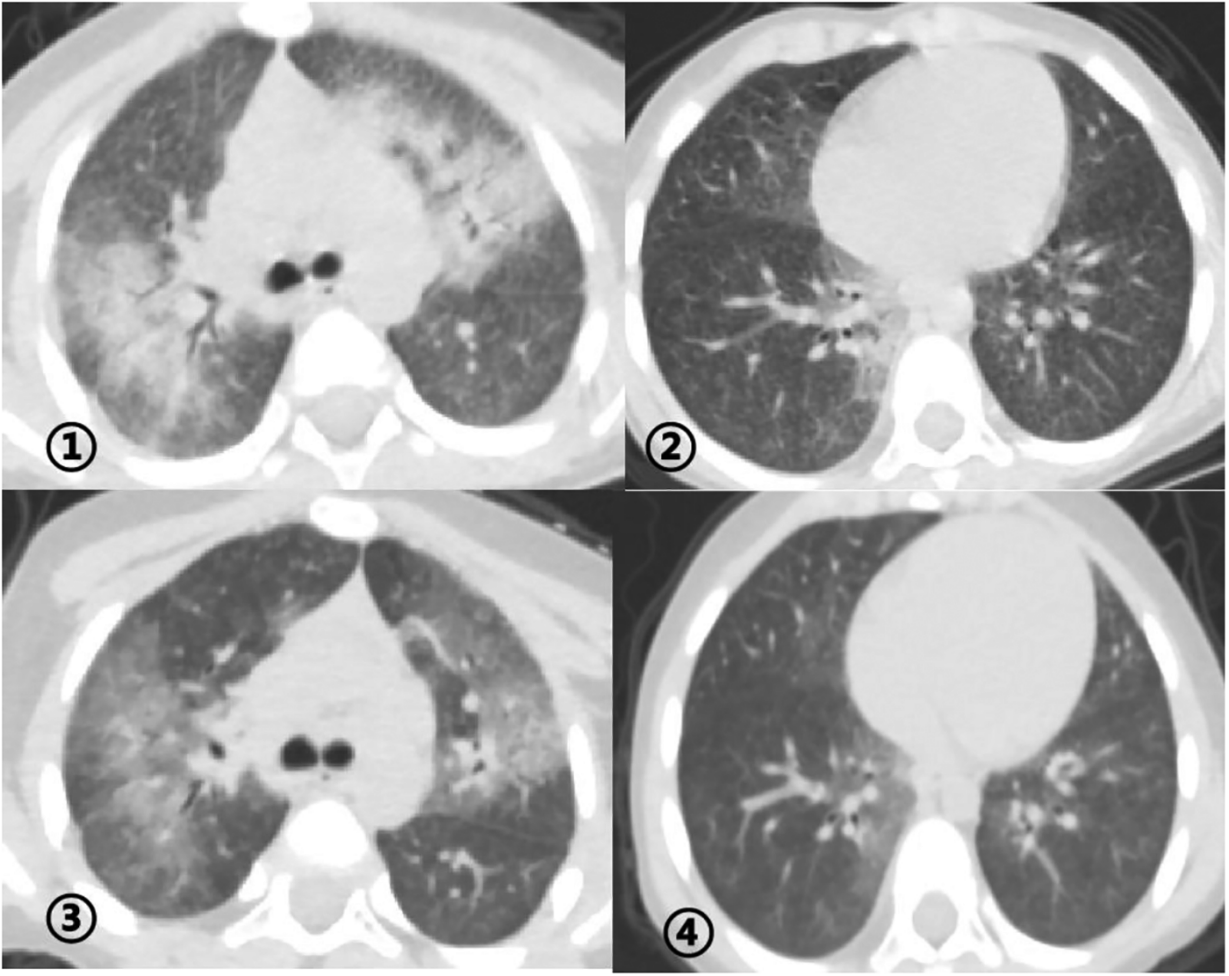
①②: Chest CT before the treatment at the age of 25-months old revealing ground glass opacities (GGO) and consolidations in both lungs. ③④: Chest CT at the age of 29-months old revealing significant absorption of GGO and consolidations in the lung after treatment for 4 months.

**Figure 4:**
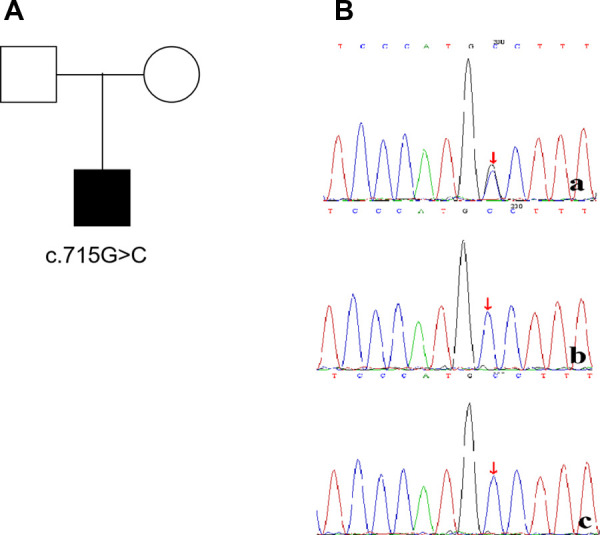
The child(**a**) has a *de novo* mutation (c.715G>C,p.A239P), his father(**b**) and mother(**c**) have no mutation at the same location.

The patient developed dry cough, shortness of breath accompanied with rashes in the following months. Physical examination showed tachypnea, retractions and clubbing fingers, as well as Chilblain-like rashes on both of cheeks (Figure 2). His respiratory symptoms revealed a lousy response to anti-infection treatment and deteriorated. Chest CT (Figure 3) showed ground glass opacities (GGO) and consolidations in both lungs, suggesting interstitial lung disease (ILD). The whole exome sequencing (WES), conducted by Beijing MyGenostics Laboratory, revealed a heterozygous variant of *COPA* gene, that is, c.715G > C (p.A239P, NM_004371), which was confirmed by sanger sequencing (Figure 4). The patient was administrated with prednisone 2 mg/kg per day for one month at the age of 25 months. His tachypnea and cough were slightly alleviated but not disappear during the treatment. Then, he received a combined treatment of sirolimus with a dose of 0.8 mg/m^2^ at the age of 26 months. Meanwhile, the dosage of prednisone gradually decreased to 0.5 mg/kg and remained unchanged. Afterwards, his cough and tachypnea gradually disappeared. Repeated chest CT at the age of 29-months old showed significant absorption of GGO and consolidations in both lungs after the 4 months of prednisone treatment and 3 months of combined sirolimus treatment. However, the patient was non-adherent to follow up.

## Discussion

*COPA* gene is located on chromosome1q23.2 and including 33 exons (54 kb). To date, most of the pathogenic COPA variants identified in patients with autoinflammatory diseases compatible with COPA syndrome are located in the 14 amino acid region and the WD40 domain with important functions, except for 4 mutations, that is c.433C > T (p.P145S) (3), c.596A > G (p.H199R) (4), c.841C > T (p.R281W) (5) and c.855G > C (p.Q285H) (6). The genetic testing of this patient revealed a variant of c.715G > C (p.A239P), located within the WD40 domain, which has been previously reported by Pamela Psarianos (7).

Clinically, COPA syndrome mainly manifests diffuse alveolar hemorrhage or interstitial lung disease, arthritis, and renal injury. Additionally, its onset is mostly in childhood, without racial predilection. Watkin et al. (8) reported that all of the patients had lung disease diagnosed as pulmonary hemorrhage, interstitial lung disease, or both. Arthritis was found in 95% of children, with the knee joint and interphalangeal joints being the most common, and rheumatoid factor positive in 43% of children. In the available literature, only 4 patients have neurological symptoms (9). Bader-meunier B et al. (6) reported a case of COPA syndrome with arthritics only. The mutation occurred on the outer surface of the adjacent blade within the seven-bladed b-propellor structure, distinctting from the previously reported hot spot mutations. To our great knowledge, there have been no previous reports of rashes in patients with COPA syndrome.

In this case, the child presented with limb weakness, and MRI of the spinal cord indicated patchy abnormal signal shadows from C1 to C6, which was consistent with acute myelitis. In terms of his clinical sympotom of vomiting, encephalitis was excluded due to no fever and convulsive seizure, normal CSF routine and biochemistry, no etiology of CSF culture and mNGS, negative antibodies against autoimmune peripheral neuropathy. Brain MRI suggested abnormal signal shadow in the medulla oblongata, area postrema syndrome was therefore taken into consideration. At the same time, according to the NMOSD diagnostic criteria formulated by the International NMO Diagnostic Group (IPND) in 2015, the child could be diagnosed as NMOSD due to positive AQP4. Additionally, this child was transiently paralyzed in his right upper limb at the age of 19 months. Combined with head MRI, the left basal ganglia encephalomalacia lesion was shown (there was no change in subsequent brain MRI), which might be episode of focal cerebral ischemia. As far as we known, NMOSD is a very rare phenotype of COPA syndrome. In the past, only one patient suffered from hearing loss due to bacterial meningitis and bilateral recurrent neuromyelitis optica. However, the presence of AQP4-Ab was not reported in this patient (10). NMOSD is an uncommon antibody-mediated central nervous system disease. Antibodies against aquaporin-4 (AQP4-Ab), a water channel expressed on astrocytes has been found in approximately 75% of patients. A recent whole-genome sequence study identified genetic variants in the major histocompatibility region that contribute to the etiology of NMOSD (11). About one quarter of AQP4-Ab positive NMOSD patients suffer from another autoimmune disease, such as systemic lupus erythematosus (SLE) and myasthenia gravis (12, 13). SLE and NMOSD share a common origin of interferonopathy. Intriguingly, Jac Williams (14) reported that either an increase in endogenous IFNα (such as in patients with SLE) or an increase in exogenous IFNα (treatment with recombinant interferon alpha) could promote the development of NMOSD. COPA syndrome is an autoinflammatory disease with autoimmune features, suggesting that the pathogenesis of COPA syndrome and NMOSD comorbidity may be similar to the SLE and NMOSD comorbidities, which may be associated with humoral immunity.

Patients with COPA syndrome shares similar clinical features with SAVI, such as ILD, joint and kidney involvement (15). Skin vasculopathy, ranging from a mild rash or livedo to severe ulcerative lesions and extensive tissue loss, is a core feature of SAVI, which is observed in 77% of the reported patients, but has yet to be described in any previous COPA syndrome patients. This is the first case of chilblain-like rashes in COPA syndrome.

At present, COPA syndrome treatment mainly refers to other autoimmune diseases. Common treatment schemes include glucocorticoids in combination with immunosuppressors such as JAK1/2 inhibitor (ruxolitinib, baricitinib). JAK inhibitors are widely used to inhibit cytokine signaling, including the downstream of interferons and other cytokines. It is reported that the application of JAK inhibitors can improve the patient's well-being and quality of life of the patients (16, 17). Glucocorticoid combined with hydroxychloroquine and mycophenolate mofetil (MMF) can also alleviate cough in COPA patients (7). In addition, Guan Y et al. (2) reported that two cases of COPA syndrome were treated with sirolimus and achieved favourable therapeutic effect. In the present report, the symptoms of the child have been remarkably alleviated after the treatment of glucocorticoid in combination with sirolimus, which supports the possibility that sirolimus may serve as an effective treatment for COPA syndrome. Blocking the mTOR pathway may be the mechanism of sirolimus in treating COPA syndrome, which is one of the downstream signaling pathways activated by STING. The long-term prognosis of COPA syndrome remains unclear due to the limited number of COPA syndrome cases and insufficient follow-up periods.

There are still several limitations in our study. Skin biopsy was not performed to verify that the chilblain-like rash in the child was vasculitis. Moreover, we only conducted short-term follow-up.

## Conclusion

In summary, we first reported a new phenotype of skin involvement in one patient with COPA syndrome, which expanded the phenotypic spectrum of this disease.

## Data Availability

The datasets for this article are not publicly available due to concerns regarding participant/patient anonymity. Requests to access the datasets should be directed to the corresponding author.
